# The changing epidemiology and clinical features of infective endocarditis: A retrospective study of 196 episodes in a teaching hospital in China

**DOI:** 10.1186/s12872-017-0548-8

**Published:** 2017-05-08

**Authors:** Wan Zhu, Qian Zhang, Jingping Zhang

**Affiliations:** 1 Department of Nosocomial Infection Control, The First Affiliated Hospital of China Medical University, No. 155 Nanjing Northern Street, Shenyang, Liaoning 110001 China; 2 Department of Infectious Diseases, The First Affiliated Hospital of China Medical University, No. 155 Nanjing Northern Street, Shenyang, Liaoning 110001 China

**Keywords:** Infective endocarditis, Clinical characteristics, Risk factors

## Abstract

**Background:**

Infective endocarditis is an uncommon but life-threatening infectious disease. To our knowledge, current investigations of the characteristics of infective endocarditis in our region are scarce. In this study, we aimed to investigate the changes in the epidemiology and clinical features of infective endocarditis.

**Methods:**

A retrospective analysis of clinical data was performed using 196 infective endocarditis cases diagnosed between June 2004 and December 2012 at The First Affiliated Hospital of China Medical University. A comparative analysis of clinical risk factors was also included.

**Results:**

The mean age of the patient cohort was 43.5 (29.0–55.8) years old. Of the 196 cases studied, 128 cases (65.3%) were left native valve infective endocarditis, 13 cases (6.6%) were left prosthetic valve infective endocarditis, and 47 cases (24%) were right valve infective endocarditis. In addition to natural valve endocarditis, many patients also exhibited various types of cardiopathy: 72 cases (36.7%) had congenital cardiovascular malformations, 37 cases (18.9%) had idiopathic mitral valve prolapse and 31 cases (15.8%) had no significant cardiac disease. The primary clinical manifestations that were observed included 168 cases with fever (85.7%), 131 cases with anemia (66.8%), 114 cases with hematuria (58.2%) and 58 cases with splenomegaly (29.6%). Positive blood cultures were detected in 96 cases (49%); the most commonly detected organism was *Streptococcus viridans* (41 cases; 42.7%), followed by 18 cases (18.8%) of *Staphylococcus aureus* and 10 cases (10.4%) of Enterococcus. With respect to risk factors, infection with *Staphylococcus aureus* or gram-negative bacilli (OR = 18.81, 95%CI = 2.39–148.2), congestive heart failure (OR = 8.854, 95%CI = 1.34–54.7), diabetes (OR = 7.224, 95%CI = 1.17–44.7) and age ≥ 60 years (OR = 6.861, 95%CI = 0.94–50.1) were major prognostic risk factors.

**Conclusion:**

Infective endocarditis is more common than previously believed, but there are regional differences with respect to the epidemiological and clinical characteristics of infective endocarditis, and its etiology is diverse and complicated. Our study of the clinically diagnosed cases of infective endocarditis verified the regional characteristics of infective endocarditis using a straightforward retrospective analysis. The geographic variations we observed in the study will be of important value to clinical diagnosis in our region.

## Background

Infective endocarditis (IE) is a relatively uncommon disease that develops on the endothelial surface of the heart. The diagnosis is traditionally based on the modified Duke criteria and rests primarily on clinical features and to a lesser extent on laboratory findings, microbiological assessment and cardiovascular imaging. If IE is not treated correctly, mortality rates are high. With the development of medical technology and pharmacotherapy, the clinical characteristics of IE have changed considerably in recent years, and the epidemiology clearly demonstrates geographical differences. To further understand the epidemiology, etiology and prognosis of IE in our region, we performed a retrospective analysis of clinical data from our hospital for the past eight years.

## Methods

### Study sample

A retrospective analysis was performed of clinical data collected from 196 cases clinically or pathologically diagnosed as IE at The First Affiliated Hospital of China Medical University from June 2004 to December 2012. The data in the retrospective analysis included general information, basic heart disease, clinical manifestations, complications and pathogenic microorganisms of IE in our hospital.

### Definition and classification of IE

IE was defined according to the modified Duke criteria [1], subject to the following two conditions: (1) confirmed by pathology, vegetation, or intracardiac abscess memory emboli with bacteria or endocardial vegetations; and (2) clinical diagnosis: meet two major criteria, one major criterion plus three minor criteria, or five minor criteria. The major clinical diagnostic criteria of IE were as follows: (1) two positive blood cultures for the same pathogen; and (2) echocardiography demonstrating endocardial involvement. Minor criteria included the following: (1) risk factors; (2) fever ≥38 °C; (3) vascular phenomena; (4) immune phenomena; and (5) positive blood cultures that do not satisfy major criterion or serological diagnosis as an IE bacterial infection. The 2009 European Society of Cardiology (ESC) issued the “Infective Endocarditis Guide” [1], which suggests the new classification method. In accordance with the infection site and whether biological or artificial materials are involved, IE is divided into four categories: left side autologous valve IE, left prosthetic valve IE, right IE, device-related IE (the latter including IE with a pacemaker or Implantable Cardiac Defibrillator (ICD) and with or without associated valvular involvement).

### Statistical methods

All data were analyzed using the SPSS 16.0 software. Univariate predictors of in-hospital death or embolic events were included in the multivariate logistic regression analyses. A *p* value less than 0.05 was considered statistically significant.

## Results

### Clinical manifestation and complications

In total, the study included 128 (65.3%) patients with left native valve infective endocarditis (NVE), 13 cases (6.6%) of patients with left prosthetic valve endocarditis (PVE), 47 patients (24%) with right heart infective endocarditis (RHIE) and 8 patients (4.1%) with device-related endocarditis. Among the 196 IE patients, the mean age was 43.5 (29.0–55.8) years old; 136 patients (69.4%) were male. The most common symptom in this group of IE patients was fever (168 cases; 85.7%), followed by anemia (131 cases; 66.8%), heart murmur (119 cases; 60.7%), hematuria (114 cases; 58.2%), splenomegaly (58 cases; 29.6%), skin and mucous membrane petechiae or ecchymoses (7 cases; 3.6%), Osler nodes (8 cases; 4.08%) and Janeway lesions (3 cases; 1.53%). A total of 40 cases (20.4%) were complicated by congestive heart failure, and renal insufficiency was present in 24 cases (12.2%).

The most common fundamental cause of IE was congenital cardiovascular malformations (72 cases; 36.7%), followed by idiopathic bicuspid mitral valve prolapse (37 cases; 18.9%) and rheumatic heart disease (19 cases; 9.7%). Among the 72 cases with congenital cardiovascular malformations, ventricular septal defect (VSD) and bicuspid aortic valve were the most common types, and atrial septal defect and patent ductus arteriosus (PDA) were rare. There were 16 cases (8.2%) each involving valvular degeneration, valve replacement surgery and diabetes. There was no significant cardiac disease in 31 cases (15.8%).

### Laboratory tests and etiology

Among these patients, 89 cases (64.5%) exhibited increased erythrocyte sedimentation rate (ESR), 109 cases (70.8%) exhibited increased C-reactive protein (CRP) and 27 cases (17.5%) were positive for rheumatoid factor (RF). There were 96 cases (49.0%) of positive blood cultures. In these 96 cases, 73 cases were positive for Gram-positive cocci, of which 51 cases were Streptococcus (41 cases were *Streptococcus viridans*, 6 cases were D hemolytic streptococcus, 2 cases were oral Streptococci, 2 cases were *Streptococcus mitis*), 18 cases were *Staphylococcus aureus* and 2 cases were *Staphylococcus lugdunensis* and Micrococcus, respectively. Gram-negative bacilli were detected in 11 cases (Loffi Acinetobacter, *Salmonella choleraesuis* and *Acinetobacter baumannii*, for 2 cases; brucellosis, *Serratia marcescens*, *Salmonella typhi*, Stenotrophomonas narrow food Aeromonas, and *Pseudomonas aeruginosa*, each type for 1 case). Ten cases were positive for Enterococci, and 2 cases were positive with fungi. Six cases involved mixed infections.

### Risk factors

Using a logistic regression analysis, we determined that age ≥ 60 years (OR = 6.861, 0.94–50.1), congestive heart failure (OR = 8.854, 1.34–54.7), diabetes (OR = 7.224, 1.17–44.7), and the presence of *Staphylococcus aureus* or gram-negative bacilli (OR = 18.81, 2.39–148.2) were the major risk factors related to death (Table 1).

**Table 1:** Death risk factor analysis in IE patients

Index	OR (95%CI)	*P*
Age ≥ 60(years)	6.861 (0.94–50.1)	<0.05
Duration ≥1 month	0.452 (0.05–3.93)	0.472
Congestive heart failure	8.854 (1.34–54.7)	<0.05
Renal insufficiency	1.350 (0.09–18.8)	0.823
Diabetes	7.224 (1.17–44.7)	<0.05
PVE	1.651 (0.17–16.3)	0.667
*Staphylococcus aureus* or negative bacilli	18.813 (2.39–148.2)	<0.05
Cardiac conduction abnormalities	2.032 (0.36–11.5)	0.422

## Discussion

IE is the infection of heart valves or the ventricular endocardium caused by bacteria, fungi and other microorganisms, such as viruses, rickettsia, chlamydia, and spirochetes. With the continuous change in the epidemiological characteristics of IE, a new set of guidelines for the management of IE was published by the Annual Congress of the European Society of Cardiology (ESC) in 2009 [1], which emphasized the diversity of clinical manifestations and risk factors (such as prosthetic valves, degenerative heart valve disease, and intravenous drug abuse) in IE. Some new additions to the guidelines are worth considering, including regional differences. Therefore, to better understand the regional characteristics of IE and to provide data to support the update of the management guidelines for IE in China, we considered it is necessary to perform a systematic study of the overall characteristics of IE in our hospital. Because our hospital is a first-class level three comprehensive teaching hospital, our data are representative of large domestic hospitals. We collected clinical data on patients with IE from the previous eight years. To our knowledge, this is the first retrospective study on IE performed in our hospital over eight years.

Among the 196 cases, there were 128 cases of NVE, accounting for approximately 65.3% of all cases. In terms of fundamental causes, the incidence of congenital cardiovascular malformations (72 cases; 36.7%) and idiopathic bicuspid mitral valve prolapse were the primary diseases, followed by no heart disease; rheumatic heart disease represented the fourth-most common underlying cause. The ranking of these causes was significantly different from that in other hospital [2]. In recent years, the rate of idiopathic mitral valve prolapse in IE has increased. A foreign study [1] reported that patients with idiopathic mitral valve prolapse are at four to eight times higher risk for IE than those with normal valves.

For the clinical manifestation, the mean age of the 196 patients was 43.5 (29.0–55.8) years old, which is younger than that in previous reports [3, 4]. Due to valvular degeneration, the elderly are more susceptible to IE. Thus, the middle-aged and elderly patients who may be considered the susceptible population should receive greater attention in our region. Fever, anemia, hematuria, heart murmur, splenomegaly, elevated inflammatory markers and a variety of peripheral vascular emboli were the primary clinical manifestations noted in the present study. Research from Polewczyk A (414 cases) [5] indicated that fever, chills and pneumonia were most likely to occur in outpatients, and the proportion of RHIE reported was significantly higher than that recorded in our hospital. Osler nodes and Janeway lesions have been relatively rare in recent years. Although anemia, splenomegaly, hematuria, peripheral vascular emboli and other indicators were not included in the diagnostic criteria for IE, the association between these symptoms and IE has been growing in recent years. In addition to fever, many complications may be considered early symptoms of IE, including heart failure, myocardial infarction, arthritis, lung infections, urinary tract infections, and cerebral emboli. These can be accompanied by other symptoms, such as palpitations, shortness of breath, lower extremity edema, joint pain, cough, sputum production, headache and numbness. Therefore, patients who exhibit an unexplained fever for more than a week in the presence of the above symptoms may be suffering from IE. Research from Spain and [6] and Jerusalem [7] revealed that more than one third of IE cases are associated with medical care, which may be related to economic development.

With respect to the etiology of IE, the proportion of IE patients who are not predisposed to heart disease and valvular degeneration is higher in western countries than in China. In this study, 49% of patients had a positive blood culture, which was significantly lower than that found by similar research by Kanafaniand [8]. This difference is primarily because patients received empiric antibiotic therapy prior to admission. Furthermore, under special culture conditions, some pathogens can be missed. In this study, the most common types of pathogen identified in IE patients in our hospital were *Streptococcus viridans*, *Staphylococcus aureus*, Enterococcus and Gram-negative bacilli. Although the level of *Streptococcus viridans* infections has declined in recent years, this pathogen remains the dominant species in our hospital. This prevalence varies considerably between domestic regions and other countries. Other results [9] from overseas indicated that Staphylococcus, especially *Staphylococcus epidermidis* and *Staphylococcus aureus*, is the main pathogen. There are regional differences in the types and distribution of pathogens. Medical care, cardiac interventional therapy, hemodialysis and intravenous drug abuse in foreign IE patients is also correlated with pathogen types [10]. Therefore, when there is a lack of clinical etiological data, an understanding of the changes in the etiology and clinical features of common pathogens may help to provide early and reasonable antimicrobial treatment for IE patients and may avoid or reduce adverse complications. Our analysis revealed that the proportion of *Streptococcus viridans* endocarditis in our hospital was higher compared with the other groups, yet that proportion of *Staphylococcus aureus* endocarditis was dominant in other countries [11]. This difference may be due to adhesion molecules from *Streptococcus viridans* and *Staphylococcus aureus* that are associated with the valvular surface or to the presence of surface adhesion factors conducive to bacterial colonization.

To further explore the impact of the major death factors, the following information was included in the analysis model: age ≥ 60 years, hospitalization ≥1 month, congestive heart failure, renal insufficiency, diabetes mellitus, new cardiac conduction abnormalities, *Staphylococcus aureus* or Gram-negative bacilli infections. Our results indicated that age ≥ 60 years (OR = 6.861, 0.94–50.1), congestive heart failure (OR = 8.854, 1.34–54.7), diabetes (OR = 7.224, 1.17–44.7), and *Staphylococcus aureus* or negative tuberculosis infection (OR = 18.81, 2.39–148.2) were major risk factors for the prognosis of IE patients. Our results exhibited some differences from other studies [4, 12, 13], in which *Staphylococcus aureus* infections, heart failure and PVE were considered major risk factors that could result in emergency surgery or death. Streptococcus and Staphylococcus were the main pathogens, whereas *Staphylococcus epidermidis*, coagulase-negative staphylococci and HACKE genus (including Haemophilus species, Aggregatibacter species, Cardiobacterium hominis, Eikenella corrodens and Kingella species) were detected less often compared to other countries [14]. The positive rate of blood culture was lower than that reported in other countries, which may be due to special culture techniques or previous antibiotic use. Thus, although the detection rate of staphylococcus is not high, its impact on patient survival cannot be ignored. Therefore, to raise the positive detection rate of pathogenic bacteria, blood culture, serology and molecular biology technology development (such as Polymerase Chain Reaction, PCR) need to be improved, as do correct collection methods and continuous inspections. Comprehensive clinical interventions may prevent the further deterioration of the heart, heart failure and heart function, which requires the cooperation of multiple subjects to improve the prognosis of IE.

## Conclusions

Our report details a large, retrospective study summarizing the epidemiology and clinical characteristics of IE of our region in China. This study is useful for the identification of clinical trends and the predictable distribution of prognostic factors. Furthermore, the death risk factor analysis underline the significance of age ≥ 60 years, congestive heart failure, diabetes, and *Staphylococcus aureus* or negative tuberculosis infection in IE patients.

## Abbreviations

CRP: C-Reactive protein
ESC: European Society of Cardiology
ESR: Erythrocyte sedimentation rate
HACKE: Including Haemophilus species, Aggregatibacter species, Cardiobacterium hominis, Eikenella corrodens and Kingella species
ICD: Implantable Cardiac Defibrillator
IE: Infective endocarditis
NVE: Native valve infective endocarditis
PCR: Polymerase Chain Reaction
PDA: Patent ductus arteriosus
PVE: Prosthetic valve endocarditis
RF: Rheumatoid factor
RHIE: Right heart infective endocarditis
VSD: Ventricular septal defect
